# OLDER CANADIAN WOMEN'S LIVING ARRANGEMENTS AND WELL-BEING DURING COVID-19

**DOI:** 10.1093/geroni/igac059.893

**Published:** 2022-12-20

**Authors:** Nicky Newton

**Affiliations:** Wilfrid Laurier University, Waterloo, Ontario, Canada

## Abstract

Living alone has been associated with lower COVID-related well-being over the course of the pandemic, although the results for women – particularly older Canadian women – depend on myriad factors. This study examines relationships between COVID-19 impact, living arrangement, and two types of well-being – psychological well-being and meaning in life – among Canadian women (N = 106) with Mage = 70.11 in two waves of data (July-September 2020 and March-May 2021). There were no differences in levels of well-being between T1 and T2, although regression analyses showed that correlates differed for type of well-being measured. Surprisingly, COVID-19 impact was only significant for psychological well-being at T1. Additionally, while levels of well-being did not differ by living arrangement at T1, by T2 both were lower for those living alone. Findings suggest adaptation to COVID-19 constraints and highlight changes in factors associated with well-being as well as the necessity of measuring well-being in multiple ways.

